# A process to control light in a micro resonator through a coupling modulation by surface acoustic waves

**DOI:** 10.1038/srep30681

**Published:** 2016-08-03

**Authors:** Guofang Fan, Yuan Li, Chunguang Hu, Lihua Lei, Yanchuan Guo

**Affiliations:** 1 Key Laboratory of Photochemical Conversion and Optoelectronic Materials, Technical Institute of Physics and Chemistry, Chinese Academy of Sciences, 100190 Beijing, China; 2 Shanghai Institute of Measurement and Testing Technology, National Center of Measurement and Testing for East China, National Center of Testing Technology, Shanghai 201203, China; 3 State Key Laboratory of Precision Measuring Technology and Instruments, Tianjin University, Weijin Road, Tianjin 300072, China

## Abstract

A novel process to control light through the coupling modulation by surface acoustic wave (SAW) is presented in an optical micro resonator. An optical waveguide modulator of a racetrack resonator on silicon-on-insulator (SOI) technology is took as an example to explore the mechanism. A finite-difference time-domain (FDTD) is developed to simulate the acousto-optical (AO) modulator using the mechanism. An analytical method is presented to verify our proposal. The results show that the process can work well as an optical modulator by SAW.

Various approaches have been developed to control light of optical waveguides in integrated optics^1^,^2^. In which, an AO interaction provides a well-established process for controlling light^3^. The AO interaction normally controls light using two fundamental structures of optical resonator and Mach-Zehnder interferometer in integrated optics^4^,^5^,^6^,^7^,^8^,^9^,^10^, in which, the frequency or transmission of a propagating light is controlled through introducing a refractive index change of optical waveguides by SAW.

In this manuscript, a novel process is developed to control light by SAW as shown in Fig. 1. During the process, the refractive index of the coupler waveguides are modulated by SAW, the modulation of the refractive index will lead to change of the coupling strength change of the coupler, the optical transmission will be modulated due to change of the coupling. Then, we realize the propagating light is controlled through a coupling modulation by SAW.

We take an optical modulator on SOI technology as an example to explore the process. A novel AO modulator with a racetrack resonator using the mechanism is designed due to a longer coupling length compared to a ring resonator^11^,^12^, in which, the refractive index modulation by SAW is analyzed, the coupling strength change of the couplers is discussed with the refractive index of the optical waveguides in the couplers. A FDTD method is performed to validate the process of the optical transmission modulation through the coupling strength change by SAW. An analytical method is introduced for further support for the novel process.

## Results

**Figure 2:**
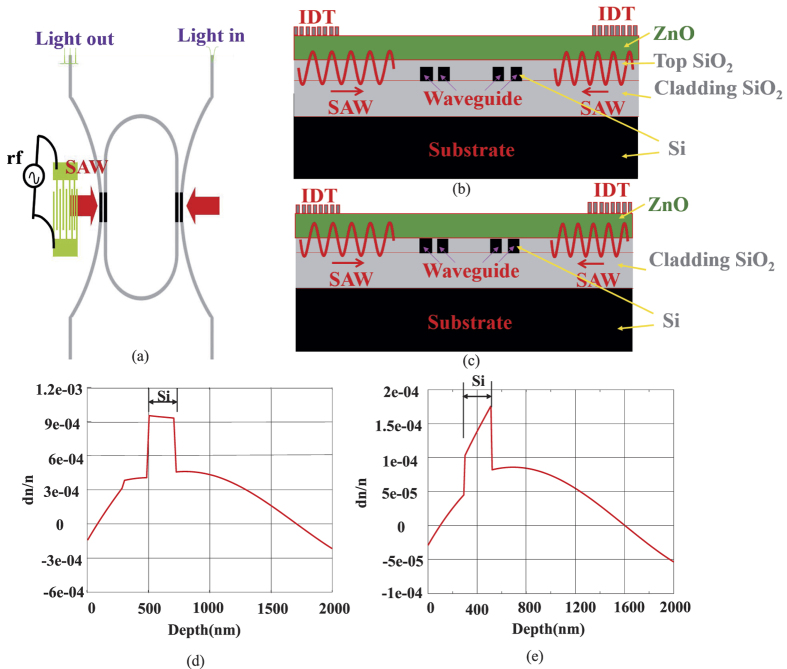
Light modulation through coupling strength modulated by SAW. (**a**) Geometry of the AO modulator. (**b**) The cross-section with a top layer of SiO2(300 nm ZnO + 200 nm SiO2 + 220 nm Si + 2000 nm SiO2 + Si substrate). (**c**) The cross-section without a top layer of SiO2(300 nm ZnO + 220 nm Si + 2000 nm SiO2 + Si substrate). (**d**) The refractive index change as a function of depth for the structure with a top layer of SiO2. (**e**) The refractive index change as a function of depth for the structure without a top layer of SiO2.

A proposed schematic of an AO modulator using the novel mechanism is shown in Fig. 2, which consist of a racetrack resonator with two waveguides for light coupling in/out and interdigital transducers (IDTs) for SAW generation. The racetrack resonator uses optical feedback, letting traveling wave interfere with itself, and the light coupling from the resonator to two waveguides leads to a transmission spectrum from input port to output port^11^,^12^. A SAW consists of a strain field propagating along the surface of a solid, which will tune the refractive index of the coupler waveguides (the black optical waveguides in Fig. 2(a)) of the racetrack resonator. Then, the coupling strength will be modulated due to the refractive index change. This will lead to the optical transmission modulation on the output port.

In order to verify the design with a top layer of SiO2 as in Fig. 2(b) can improve the acousto-optical effect of silicon waveguides, we design the structures with a top layer of SiO2(the depth of layers: 300 nm ZnO + 200 nm SiO2 + 220 nm Si + 2000 nm SiO2 + Si substrate) in Fig. 2(b) and without a top layer of SiO2 (the depth of layers: 300 nm ZnO + 220 nm Si + 2000 nm SiO2 + Si substrate) in Fig. 2(c). Based on equ. (1), we calculate the refractive index change by SAW for different layers as shown in Fig. 2(d,e) for the structures with and without a top layer of SiO2, respectively. One can observe that the optical waveguide on SOI technology can have a strong modulation by introducing a ZnO layer, although the silicon waveguide is non-piezoelectric. The modulation increases with the depth closer to silicon waveguide due to a stronger optical field of the silicon waveguides.

Compared with Fig. 2(d,e), one can find that an AO structure with a top layer of SiO2 shows better uniformity and larger modulation for the refractive index on the silicon waveguide than a structure without a top layer of SiO2. This means that an introduction of SiO2 layer between ZnO layer and silicon waveguides can improve the modulation of silicon waveguide by SAW largely. According our design, the simulation shows that the refractive index change on a Si optical waveguide is about 10^–3^ for an AO device with a top of SiO2 layer under a SAW power density of 200 w/m as shown in Fig. 2(d).

**Figure 3:**
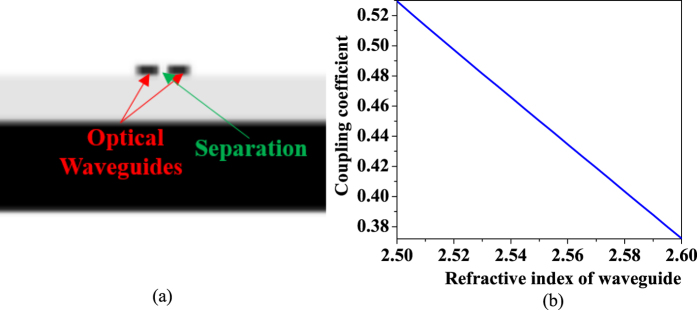
Coupling strength of two straight waveguides with the effective index change. (**a**) Geometry of the two coupling waveguides (the width of the optical waves is 500 nm and the separation is 180 nm), (**b**) Coupling strength as a function of refractive index change.

In the above discussion, the refractive index modulation of optical waveguides by SAW have been analyzed. Here, the coupling strength modulation due to refractive index change will be discussed. A FDTD method is performed to evaluate the relationship of coupling strength and refractive index as shown in Fig. 3(b). One can observe that a coupling strength changes with the refractive index of two adjacent waveguides. This means the coupling strength can be modulated by changing the refractive index of two adjacent waveguides. Hence, the coupling strength can modulated through changing the refractive index of the optical waveguides by SAW.

## Discussion

Based on the above discussion, the refractive index of optical waveguides can be changed by SAW. The change of refractive index will lead to the coupling strength modulation. In other words, the coupling strength can be modulated by SAW. Then, we will discuss that the modulation of coupling strength will lead to change of optical transmission.

In our discussion, a design of an AO modulator is shown in Fig. 2(a) with cross-section of Fig. 2(b), in which, the width of optical waveguides is 500 nm, the radius of the inner ring is 5 μm, the separation of in/output waveguides and the racetrack resonator is 180 nm, the coupling length of optical waveguides is 3.5 μm. A FDTD is performed to simulate the AO modulator on the racetrack resonator, in which, the refractive index change is assumed as to be 0.0025 by SAW.

**Figure 4:**
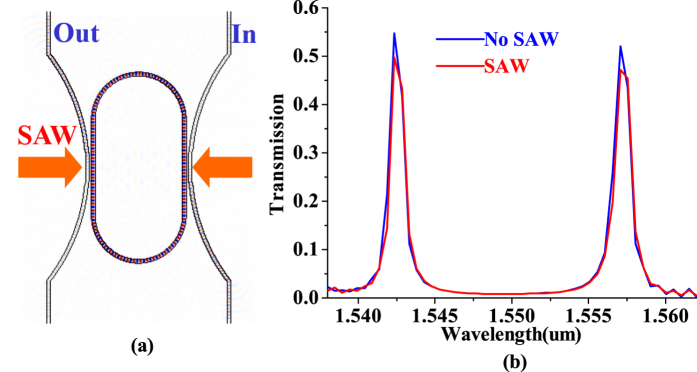
Simulation of the AO modulator on the racetrack resonator using the novel mechanism with and without SAW modulation. (**a**) Simulation using FDTD. (**b**) Transmission change due to SAW modulation.

Figure 4(a) shows the optical travel image of the racetrack resonators using FDTD simulation. The strong color along the racetrack resonator means the optical waves travel and interfere with itself using optical feedback in the racetrack resonator, which mean the racetrack resonator has a high quality factor. Also, one can find a weak color in the output port of light, this shows that the optical waves of the resonator is coupled to the output waveguide and leads to a transmission. The transmission for different optical wavelength is shown as the blue lines in Fig.4(b), in which, the two peaks means that the resonator has two resonance.

When SAW works on the racetrack resonator, the coupling strength of couplers will change due to the refractive index modulation by SAW. This will lead to modulation of the optical transmission for the optical racetrack resonator as the red lines of Fig. 4(b). Compared with the red and blue lines for the transmission, one can observe that the optical transmission of all two resonances is reduced by about 10% due to SAW modulation with a SAW power of 100 mw. This means that the optical racetrack resonator can be modulated by SAW using this process. In other words, the proposal design can work well as an acousto-optical waveguide modulator.

**Figure 5:**
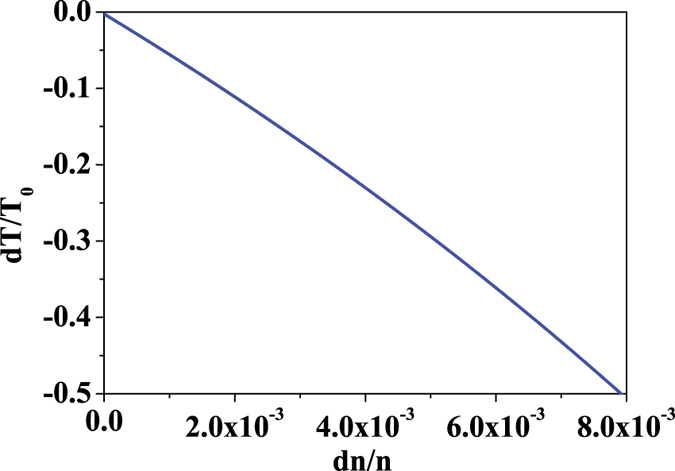
The relationship of the transmission with refractive index from an analytical method for the design (the parameters are from FDTD simulation).

Moreover, an analytical method is introduced to verify our proposal. According to the method, the transmission change with the coupling (*k*) on the Eq.(2), which means that our process can work to modulate the transmission through the coupling change by SAW. The relationship between the transmission and refractive index of the coupling waveguides is plotted as shown in Fig. 5, in which, the relationship of the coupling (*k*) and refractive index (*n*) is gotten from Fig. 3(b) through the above analysis using FDTD.

One can observe that the optical transmission change with the refractive index because the coupling (*k*) is changed due to the refractive index modulation by SAW. Hence, this means that our process can work as an optical modulator by coupling change through the refractive index modulation using SAW. In addition, there is a difference of the transmission change for index change of 0.0025 between FDTD and the analytical method because the analytical method is a rough research. Also, one can find that the transmission modulation range is decided by change of refractive index, which is determined by SAW power.

We have proposed a novel process to control lights using SAW is developed. An AO modulator using the novel process is designed on the racetrack resonator. The refractive index change by SAW is studied in the design. The coupling strength of cavity and in/output waveguides is discussed with refractive index change. The novel design of an AO modulator is simulated using FDTD. The simulation results show that the novel mechanism can work well as an AO modulator. Also, an analytical method is introduced to verify the design.

## Methods

### The modulator design using the novel AO process

In our design, IDT electrodes for SAW generation are placed on top of a piezoelectric ZnO layer due to a Si waveguide without piezoelectricity, from where, the generated SAW propagates to the rest of the sample along the ZnO layer^13^. The positions of IDTS are designed to create an acoustic cavity, in which, SAW can reflect on IDTS. This will increase the acoustic fields. The optical waveguides are placed on the acoustic field antinodes, and the distance between IDTs and the center of Si waveguide should be as a multiple of SAW wavelength in order to have a maximum modulation for the refractive index of Si waveguides. The widths of Si waveguides are much smaller than a SAW wavelength, so the SAW-induced refractive index change can be considered as a constant across the optical waveguides.

Under the ZnO layer, a SiO2 layer is designed on the top of optical waveguides in order to reduce acoustical reflections on optical waveguides and the optical waveguide losses. More important, a SiO2 layer on the optical waveguides can increase the refractive index modulation by SAW, compared to a structure without a top of SiO2 layer.

When the optical waveguide is modulated by the SAW, the amplitude of refractive index change due to SAW modulation can be described by the equation^7^,^14^:


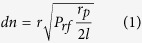


where *r* is acousto-optical coupling factor, which is determined by the photoelastic coefficients, optical field distribution of the waveguide, and SAW strain fields. *P*_*rf*_ is radio-frequency (rf) power, *r*_*p*_ means *P*_*rf*_ converted to the acoustic mode, *l* means the effective length of SAW in the active region.

### Coupling strength modulation of the resonator and in/output waveguides

In our design, the control of light by SAW is directly decided by the coupling strength modulation of the resonator and in/output waveguides. In which, the coupling strength is modulated due to the refractive index change of the coupler waveguides by SAW modulation. An optical structure shown in Fig. 3(a) is introduced to evaluate the relationship of the coupling strength with the refractive index of the two optical adjacent waveguides, in which, the width of the optical waves is 500 nm and the separation is 180 nm.

### An analytical method for the optical resonators

Moreover, an analytical method is introduced to verify our proposal^12^, the transmission of an optical resonator can be expressed as the followed^12^:


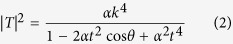


where *k*, *t* are coupling and transmission coefficient of the coupler (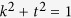), *α* are losses of a micro-resonator, 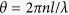 is phase of a resonator (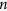 is refractive index of optical waveguides, 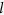 is perimeter of the resonator and 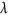 is optical wavelength).

## Additional Information

**How to cite this article**: Fan, G. *et al*. A process to control light in a micro resonator through a coupling modulation by surface acoustic waves. *Sci. Rep.* **6**, 30681; doi: 10.1038/srep30681 (2016).

## Figures and Tables

**Figure 1:**
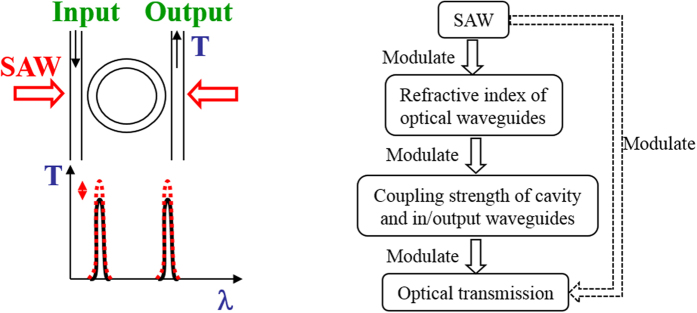
A novel process to control the light by SAW.
